# Exploring the Potential of Natural Product-Based Nanomedicine for Maintaining Oral Health

**DOI:** 10.3390/molecules27051725

**Published:** 2022-03-07

**Authors:** Rajeev Kumar, Mohd A. Mirza, Punnoth Poonkuzhi Naseef, Mohamed Saheer Kuruniyan, Foziyah Zakir, Geeta Aggarwal

**Affiliations:** 1 Department of Pharmaceutics, School of Pharmaceutical Sciences, Delhi Pharmaceutical Sciences and Research University, Sector-3, M.B. Road, PushpVihar, New Delhi 110017, India; rajeevroy218@gmail.com; 2 Department of Pharmaceutics, School of Pharmaceutical Education and Research, Jamia Hamdard, Hamdard Nagar, New Delhi 110062, India; aamir_pharma@yahoo.com; 3 Department of Pharmaceutics, Moulana College of Pharmacy, Perinthalmanna 679321, India; drnaseefpp@gmail.com; 4 Department of Dental Technology, College of Applied Medical Sciences, King Khalid University, Abha 61421, Saudi Arabia; mkurunian@kku.edu.sa

**Keywords:** dental diseases, essential oils, herb, natural products, nanotechnology, regulations

## Abstract

Oral diseases pose a major threat to public health across the globe. Diseases such as dental caries, periodontitis, gingivitis, halitosis, and oral cancer affect people of all age groups. Moreover, unhealthy diet practices and the presence of comorbidities aggravate the problem even further. Traditional practices such as the use of miswak for oral hygiene and cloves for toothache have been used for a long time. The present review exhaustively explains the potential of natural products obtained from different sources for the prevention and treatment of dental diseases. Additionally, natural medicine has shown activity in preventing bacterial biofilm resistance and can be one of the major forerunners in the treatment of oral infections. However, in spite of the enormous potential, it is a less explored area due to many setbacks, such as unfavorable physicochemical and pharmacokinetic properties. Nanotechnology has led to many advances in the dental industry, with various applications ranging from maintenance to restoration. However, can nanotechnology help in enhancing the safety and efficacy of natural products? The present review discusses these issues in detail.

## 1. Introduction

Dental diseases are a major public health concern and they severely impact the quality of life of individuals. They represent a very important health problem in several countries and create distress among individuals during their lifetimes, causing pain, uneasiness, deformity, and even death. According to WHO, oral diseases affect approximately 3.5 billion people globally (https://www.who.int/news-room/fact-sheets/detail/oral-health, accessed on 5 January 2022).

The most common dental diseases are dental caries (tooth decay), oral cancer, periodontitis (gum disease), noma, and trauma to the oral cavity. Globally, oral cancer is the most prevailing type of cancer. Additionally, with the increase in the consumption of processed and sweet foods, high in free sugars, such as chocolates, candies, and other confectionaries, the problem has worsened. Children are more exposed to this seriousproblem. Soft drinks come into contact with the surfaces of the teeth, causing demineralization. Chewing gums slowly release sugar content in the mouth, which promotes tooth decay. Moreover, diseases such as obesity, diabetes, cancer, chronic respiratory conditions, and cardiovascular complications are also associated with oral diseases. Furthermore, high consumption of tobacco and alcohol also contributes to dental problems. The human mouth is already home to several bacteria, fungi, viruses, and protozoa species, which together constitute the oral microbiome. These microorganisms are determinants of oral health, and infection occurs when the equilibrium is interrupted, which allows the invasion of pathogens [1]. Moreover, consumption of a high-carbohydrate diet disturbs the acid mantle in the oral cavity. The microorganisms convert the carbohydrates into acids, which degrades the hydroxyapatite in the tooth enamel. This promotes contamination with bacteria and the formation of dental caries [2,3]. Nowadays, people are more prone to oral diseases because they remain indoors, which causes vitamin D deficiency. Given that vitamin D is associated with the absorption of calcium, a lack of this vitamin can lead to hypoplasia, which can also contribute to dental caries [4]. Developing and underdeveloped nations are more prone to such problems due to poor health hygiene, lack of awareness, and improper health facilities. It is also a fact that dental treatment is expensive in developed countries, accounting for approximately 5% of the total health expenditure, which is mostly borne by the individuals [5]. Therefore, with current lifestyle choices, maintaining oral hygiene is essential and cannot be neglected.

It is believed that with traditional diet practices (with low sugar content), most of the dental diseases can be avoided [6]. Further, doctors advise the use of fluoride-based mouthwashes, toothpastes, and gels to prevent dental caries [7,8]. However, synthetic products should not be used in the long term. Overuse can cause oral or systemic adverse reactions such as irritation, swelling, itching, and dry mouth [9]. Many over-the-counter (OTC) medications contain ingredients such as chloral hydrate, nitrites, etc., which are consumed by oral pathogens and release products, which causes halitosis [10]. Long-term use of antiplaque agents has been known to be associated with staining of teeth and taste alterations. Furthermore, dental infections are progressively linked with the formation of biofilms. Bacterial/fungal biofilms promote drug resistance against antimicrobials, which makes the infection difficult to treat. Additionally, many challenges, such as side effects/adverse reactions and poor bioavailability issues, may lead to withdrawal because of the inconvenience of long-term therapy.

Herbal products have been used since antiquity for the prevention of diseases and to promote well-being. The Vedic age in India documented the use of herbal remedies in Rigveda and Charaka Samhita [11]. The use of twigs from the *Salvadora persica* tree (known as miswak) for teeth cleaning was reported 7000 years ago in Arabic culture. Studies have proven that miswak possesses antibacterial activity, which prevents the formation of dental plaque (https://clinicaltrials.gov/ct2/show/NCT04561960, accessed on 5 January 2022). In, 1986, miswak was recommended by WHO for oral hygiene. Following this, extracts from *Salvadora persica* were added to toothpastes. Ayurvedic texts also mention the traditional practice of oil pulling. A teaspoon of coconut oil, when swirled in the mouth for around 10–20 min, is believed to improve oral health [12]. Similarly, clove oil has been used for centuries as an analgesic for toothache.

However, with the progression of science, evidence has become a problem for herbal remedies. For this reason, herb-based natural treatments were confined to only a few regions of the world where they have been practiced for a long time, although, with categories such as dietary supplements, neutraceuticals, and botanicals, herbals could be placed into the market. Therefore, now, with the availability of sophisticated technologies and regulatory guidelines, healthcare companies are beginning to take advantage of the opportunities associated with herbal products. The importance of herbal products in the pharmaceutical industry can be demonstrated by the fact that 50% of the drugs approved during the last 20 years were derived from plant sources [13]. Due to cost-effectiveness, cultural acceptability, and minimal adverse drug reactions, 75–80% of the world population relies on herbal drug products. Thus, the paradigm in oral healthcare is also witnessing a shift towards herbal remedies. Presently, various organizations across the world, including WHO, are promoting herbal products for better health. In fact, developed countries have also embraced herbal products as complementary and alternative medicine (CAM) [14]. Herbal medicines are supposed to be safe if not adulterated and quality standards are maintained. With the increasing awareness of the effectiveness and benefits of herbal products, financial aid is also being offered by different research supporting bodies. Nevertheless, this potential has not been exploited to the maximum. It cannot be ignored that even herbal products present some shortcomings. This review details these limitations and discusses the strategies that can be adopted to improve their acceptability in the dental care product industry.

## 2. Herbal Remedies for Dental Diseases

A great deal of research has been carried out that proves the activity of herbal ingredients against several dental diseases. Rosemary and *Bougainvillea glabra* essential oil show anti-inflammatory activity that is modulated by the inhibition of histamine and prostaglandin signals [15,16]. This suggests that essential oils with anti-inflammatory activity can be used for the treatment of gum diseases [17].

Treatment of dental diseases often requires topical antioxidants in the form of toothpastes, gels, and mouth rinses. There are numerous factors, such as stress, disease, or dental procedures, that can increase the levels of free radicals; bacterial infections also trigger immune responses, which add to free radical formation. Prolonged infection can result in inflammation, which, if left untreated, can lead to chronic stress. Although salivary anti-oxidants can control free radicals, this is often insufficient during oral/systemic infection. Therefore, additional antioxidant supplements are required to fight inflammation [18]. Consumers are now becoming aware of the harmful effects of synthetic antioxidants. Essential oils from rosemary and lavender were tested for their IC_50_ values, which demonstrated their antioxidant activity [19].

A clinical trial study was conducted on 60 subjects, where the antimicrobial effect of neem extract was investigated. It was found that liquid neem extract significantly (*p* < 0.05) reduced the *Lactobacillus* and *S. mutans* counts, thus suggesting activity against gingivitis and dental plaque [20]. In another study, the antimicrobial effect of Triphala powder against *S. mutans* was tested. The results showed complete inhibition of bacterial growth in 6 min with an MIC of 3.125 mg/mL, which was comparable to the MIC of 0.2 µg/mL exhibited by 0.2% chlorhexidine [21]. Thomas et al. [22] proposed that mouthrinses containing extracts of garlic and lime have significant antibacterial and antifungal activity against lactobacilli, *S. mutans* (*p* = 0.001), and *C. albicans* (*p* < 0.001). Chlorhexidine and fluoride are the main constituents of chemical-based mouthwashes due to their antibacterial activity. The study showed the effective antimicrobial activities exhibited by herbal ingredients when compared with synthetic mouthwashes, suggesting their potential to be used as a substitute for synthetic mouthwashes. Some authors have claimed the anti-cariogenic potential of dentifrices containing clove oil, extracts of black pepper, mint, long pepper, pomegranate, babool, and miswak [23,24]. Most essential oils have demonstrated antimicrobial properties, which is the reason for the rise in their popularity in the treatment of dental infections. A significant amount of research has been carried out to prove that the MIC values of synthetic antibacterial agents are considerably reduced by different essential oils. Their antimicrobial activity has been demonstrated against both Gram-positive and Gram-negative bacteria, fungi, and yeasts [25]. For this reason, many oral hygiene products contain mixtures of essential oils, which serve as antimicrobial agents, control bad smells, and reduce oral bacteria. In another study, a product containing peppermint oil, lemon oil, and tea tree oil was used to treat bad oral smell in 32 intensive care unit patients. After 5 min of essential oil treatment, the strength of the bad smell was significantly lowered. This study showed that, besides the antimicrobial activity, essential oils can also control bad oral smell [26].

According to a report, herbal ingredients from clove, miswak, neem, propolis, and aloe vera exhibit multiple activities, such as anti-inflammatory, antibacterial, antioxidant, and so on, which suggest their role in the treatment of dental plaque and gingivitis [27].

Additionally, herbal formulations have the advantage of being sugar- and alcohol-free. Natural sweeteners such as stevia extracts and xylitol are added to prevent the problem of halitosis [24].

There are increasing numbers of reports that suggest the biofilm disruption activity of herb extracts [28]. In a study by Ramalingam et al. [29], mixtures of *Acacia arabica* and triphala extracts were tested for their biofilm disruption activity against *A. viscosus*, *C. albicans*, *L. casei*, and *S. mutans*. The results revealed that the extracts, at a concentration of 150 µg/mL, not only reduced the biofilm by 91–99% but also prevented bacterial adhesion, thus stressing that they can act as effective anti-caries agents. Nonetheless, even essential oils have recorded biofilm disruption activity. For instance, in a study, the activity of *Allium sativum* essential oil was tested against fluconazole-resistant *C. albicans* biofilms. It was found to be effective at a concentration of <1 mg/mL, which suggested its possible use to prevent denture stomatitis [30]. A similar study carried out suggested the possible role of *Cymbopogon citratus* essential oil against polymicrobial biofilms. The study proved its inhibitory and cytotoxic activity against different species responsible for dental caries, with the added advantage of inhibiting the adhesion of biofilms to dental enamel [31].

Among dental disorders, oral cancer is the major cause of death worldwide. Considering the toxicity of anticancer agents coupled with the emergence of resistance, it has become imperative to search for low-risk therapies for cancer treatment [32]. Studies have suggested that *Lawsonia inermis* essential oil has the potential to be used as an adjuvant in cancer treatment [33]. In another study, a cocktail of extracts of *Ganoderma lucidum*, *Antrodia camphorata*, and Antler showed an IC50 of 15 mg in 72 h during an MTT assay. Further, it inhibited the proliferation and migration of cancer cells without any toxicity/adverse events [34]. Curcumin causes apoptosis of cancer cells via the production of reactive oxygen species and suppression of p53 protein [35]. Simultaneously, curcumin has been found to possess anti-inflammatory and antioxidant properties, which are modulated by preventing lipooxyenase- and cyclooxygenase-mediated inflammation [36]. This property acts synergistically in cancer treatment. Research has shown that *Cryptomeria japonica* essential oil induces apoptosis of human oral epithelial carcinoma cell lines such as KB cells, which may suggest its potential as a chemotherapeutic agent [37]. In a similar study, *Thymus caramanicus* essential oil has shown anti-proliferative and cytotoxic properties on KB cells [38]. Another recent study has shown that essential oil from *P. rivinoides* exhibits cytotoxic activity in oral squamous cell carcinoma cell lines [39]. Additionally, it has been shown that herbal ingredients not only act as chemopreventive and chemotherapeutic agents but also have beneficial effects on chemotherapy-induced side effects. Herbal medicines such as Rikkunshito, Hangeshashinto, and Goshajinkigan have been known to ameliorate side effects such as oral mucositis, diarrhea, anorexia, neurotoxicity, etc. [40].

The many benefits associated with herbal remedies have promoted the use of herbal-based products in the oral health industry.

The potential applications of different essential oils and herbal ingredients investigated for different dental diseases are enumerated in Table 1.

In addition to standalone products, herbal ingredients can be used in synergistic combinations. This approach is known as “herbal shotgun” [41]. For instance, mixtures of extracts of neem, aloe, eucalyptus, hibiscus, rose, and tulsi are useful for the inhibition of most periodontal pathogens and the treatment of dental caries [42]. It is believed that this strategy can offer a multi-targeted effect with maximum benefits and lower potential to develop drug resistance.

However, regarding natural products, there are many shortcomings that cannot be ignored. The dental industry must address these in order to succeed in the global sector.

## 3. Challenges of Herbal Therapies

**Figure 1:**
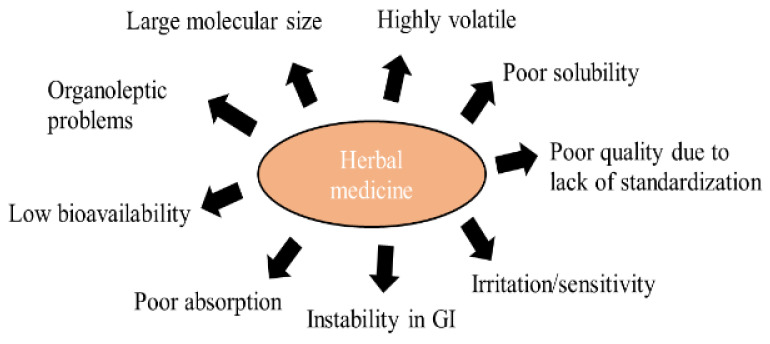
Limitations of herbal medicines restricting application in dental industry.

Herbal products are now widely present in the market in different regulatory categories. They are also becoming in-demand products for primary healthcare treatment over the conventional medicinal system, due to their fewer side effects and better acceptance. Despite the many advantages, the delivery of herbal ingredients is a challenge (Figure 1), which is discussed in a subsequent section. For instance, essential oils are volatile in nature, which limits their application. Further, when used topically, they can cause irritation/sensitivity to the oral mucosa, which restricts their use [25]. Consequently, other challenges of herbal therapies, such as low solubility, low permeability, long duration of treatment, poor bioavailability, and other challenges (discussed in subsequent sections), limit their potential.

### 3.1. Safety Issues with Herbal Products

As we have discussed, medicinal plants contain many potential ingredients that can be used to treat oro-dental diseases. Due to the long history of effectiveness of herbal ingredients against dental diseases, people use them without caution. It is generally believed that herbal remedies are relatively safe when compared to allopathic treatments. However, the claim that herbal medicine does not have any toxic or side effects is not true in all cases. Allergic reactions to essential oils cannot be ignored. Studies have shown that essential oils from sandalwood, lavender, tea tree, and clove are most likely to cause irritation and inflammation. The principle components responsible are benzyl alcohol, geraniol, eugenol, hydroxyl-citronellal, etc. Subsequently, the use of high concentrations/doses of essential oils can trigger adverse reactions [26]. Various factors, such as the amount of biological content, source of the material, and the route of exposure, should also be taken into consideration with regard to irritation potential.

The latest research studies show that extracts of herbal preparations may have adverse/side effects even if the preparation is used in low doses. There are a few plants reported in research studies that are well known for their medicinal value and are currently used for treatment but exhibit toxic effects too [58]. Moreover, synergistic combinations of herbal ingredients are used for better therapeutic outcomes, which would mean that the content of chemical constituents may be several times greater and thus linked with increased risks of toxicity (“National policy on traditional medicine and regulation of herbal medicines”, Report of a WHO global survey, World Health Organization, 2005). However, increased side effects do not mean that the use of herbal medicinal preparations should be avoided. Judicious use can be ensured by pharmacological screening and evaluation of the components in the preparation [58].

### 3.2. Patient Acceptance

Although the good therapeutic efficacy of herbal products has been demonstrated, patient acceptability is another important criterion that cannot be overlooked. Since the product is meant for the treatment of dental diseases, good taste and smell, besides other organoleptic properties, are also essential. Essential oils cannot be ingested orally, and can only be used for local application in the form of gargles, mouthwashes, and ointments.

The main problem with essential oils is their strong odor. Tea tree oil (*Melaleuca alternifolia*) has shown antimicrobial properties when tested in 34 patients. However, when the organoleptic properties were tested against Colgate toothpaste, an unpleasant taste was experienced [59]. Similarly, mouthwashes containing tea tree oil have exhibited poor taste and a stinging sensation in the mouth [60]. Although most of the essential oils, due to their strong smell, are used to mask odor in oral diseases, they are nonetheless often not accepted by the consumers. Eucalyptus oil and tea tree oil are more commonly known essential oils with a strong odor that are poorly acknowledged [26].

### 3.3. Poor Bioavailability

During the formulation of herbal drug products, the permeability of drug molecules across the epithelial mucosal barrier must be achieved for better therapeutic action of the drug product. Variations in the permeability of a drug across different locations in the oral mucosa can be observed. The keratinized regions contain ceramides, which act as a barrier for hydrophilic drugs, whereas non-keratinized areas limit the permeation of hydrophobic drugs.

The washing action of saliva in the mouth also contributes as a barrier against adequate delivery [61]. Further, the instability of herbal active compounds in the gastric region cannot be ignored.

Most of the herbal constituents isolated are hydrophobic in nature, and thus poorly soluble, making them less bioavailable, which needs to be taken into account for efficient therapeutic action [62]. This would mean that higher doses will be required, which can result in adverse effects and poor patient compliance. Additionally, phenolic-based plant constituents are water-soluble, which restricts their absorption across the lipid membrane. Further, improper molecular size is again a challenge thatcontributes to poor absorption. Chinese medicines comprise larger molecules that are difficult to absorb and this affects other phyisco-chemical attributes [63]. On the other hand, regarding essential oils, although they are small molecules that are able to permeate and absorb, the faster metabolism and short half-life lead to low bioavailability [64]. Many of the marketed products, such as curcumin and ellagic acid, have poor bioavailability because of their lower solubility in aqueous media and extensive metabolism. In a study carried out on rats, no curcumin was found in biological fluid/plasma when 400 mg of curcumin was administered via the oral route; however, a very small amount of curcumin was found in the portal blood [65].

It is for these reasons that most of the plant-based drugs have shown promising potential during invitro studies but under-perform in the clinical stage due to poor bioavailability.

### 3.4. Long Duration of Treatment

In most herbal medicinal products, the short duration of action represents a major limitation. Formulation scientists have to keep in mind that the dosing frequency of the dosage form should be minimal. Scientists are still working on improving the duration of action as well the onset of action of herbal medicinal products.

### 3.5. Lack of Harmonized Regulations

Herbal products are marketed in different product categories in different parts of the world. Currently, many regulatory categories exist for herbal medicinal products thatcomprise over-the-counter drugs, prescription drugs, traditional medicinal products, and dietary supplements. There is a need for the establishment of strict global and regional regulatory mechanisms for the monitoring of herbal medicinal products [66]. The magnitude of quality, safety, and efficacy data requirements for product registration varies from region to region. There should be a harmonized data requirement throughout. Furthermore, most of the herbal products available in the market lack evidence of their safety and efficacy. Improper cultivation and harvesting techniques and improper storage conditions create an urgent need to standardize herbal preparations. Another problem is contamination with heavy metals, which occurs during the cultivation stage. Adulteration of herbal ingredients is also a major quality concern.

WHO has been pioneer in setting the parameters for the quality, safety, and efficacy of herbal medicinal products to meet the basic criteria for evaluation. A set of basic parameters for the evaluation of herbal drug products have also been added in pharmacopeial monographs. Scientists are still performing research on herbal medicines to deliver them with maximum bioavailability and concentration to target cells [65]. Therefore, a suitable delivery system has to be developed to realize the full potential of natural products.

## 4. Nanotechnology in Herbal Dentistry

Although herbal ingredients have shown extensive potential in the treatment of dental diseases, one of the major limitations is their unfavorable physicochemical and pharmacokinetic properties, which contribute towards inadequate performance. Another problem is their instability in the biological milieu. Furthermore, the physical stability of active compounds cannot be overlooked. Environmental conditions, processing, and handling of plant materials can lead to degradation due to oxidation and dehydrogenation reactions, which ultimately affect the organoleptic properties [67].

**Figure 2:**
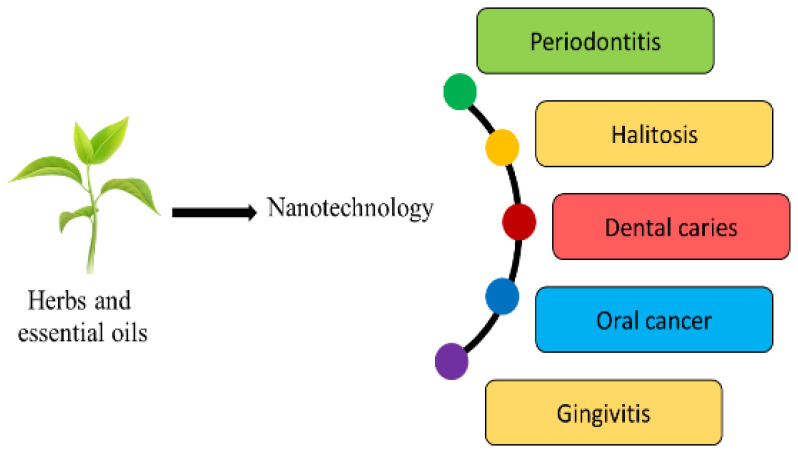
Applications of nano-herbal technology in diverse dental domains.

Many approaches have been used to enhance their absorption, stability, and pharmacokinetic profile. One suitable method would be to encapsulate the phytoconstituents in a suitable carrier system, which will help in realizing the full potential of the herbal active moiety (Figure 2). The solubility profile can be improved by forming salts with weak acids/bases. However, the salt formation technique cannot be applied to all the phytoconstituents.

Nanotechnology has already produced some promising outcomes in the delivery of phytoconstituents. The technique has been found useful to assist in overcoming low systemic bioavailability and inadequate solubility. The drug delivery potential of nanoformulations has received a great deal of attention recently, with polymeric nanoparticles and lipid-based delivery systems such as phytosomes, ethosomes, liposomes, transferosomes, and nano-emulsions all attracting much interest [68].

### 4.1. Nanotechnology to Enhance Solubility of Natural Bioactives

It is well proven that reducing the size of the herbal bioactives can enhance the solubility and dissolution. Depending upon the intended site of action, the size of the formulation can be regulated to facilitate transport across the biomembrane. Since the bioavailability of a poorly soluble drug is limited by dissolution, even a minute increment in solubility will have a significant impact on the bioavailability [69]. For instance, curcumin, with very good anti-inflammatory activity if used as a powder or in other conventional delivery systems, shows low oral absorption due to its hydrophobic nature. Therefore, nanomicelles were prepared, which entrapped curcumin in a hydrophobic core, rendering them miscible with water. The delivery system enhanced the solubility, which proved to be successful in reducing inflammation in gingivitis and mild periodontitis [70]. Various approaches, such as the formulation of nanosuspensions, nanoemulsions, nanocrystals, etc., have been used, where the particle size of the delivery system is reduced, which ultimately enhances the solubility/dissolution (Table 2).

### 4.2. Nanotechnology to Enhance Permeability of Natural Bioactives

There are a number of ways through which the permeation of herbal ingredients can be facilitated. Surface coating with hydrophilic surfactants/polymers or otherwise lipophilic polymers can be done to assist the transport if the hydrophilicity/lipophilicity of the molecule is a barrier. Consequently, mucoadhesive formulations can be prepared by using bioadhesive polymers. This will enhance the residence time of the formulation in the oral cavity, which will provide ample time for sufficient permeation. Further, encapsulating the herbal active moiety into a nanodelivery system will not only ensure better permeation but also provide stability to the molecule [89]. Herbal extracts from *Trypterygium wilfordii* have shown good potential as anticancer agents, but they exhibit insolubility and poor intestinal absorption. Lipid-based nanocarriers such as lipid nanoparticles [90] and lipid nanospheres [91] were developed to enhance the solubility and permeability. In another study, phospholipid-based phytosomes functionalized with protamine and loaded in chitosan sponges were prepared. The delivery system provided mucoadhesive properties coupled with enhanced permeation through the buccal mucosa to provide a 244% increase in bioavailability [92]. Tonglairoum et al. [93] reported the complexation of clove oil and betel oil with cyclodextrins to enhance the solubility. It was further incorporated into nanofibers, which provided fast release of the oil and enhanced the antifungal activity against Candida sp. The study proved that the formulation can be useful for the treatment of denture stomatitis.

### 4.3. Nanotechnology to Enhance the Therapeutic Performance of Natural Products

The concept of utilizing nanotechnology to enhance the therapeutic performance is not new. There are reports that the nanosizing of the formulation enhances the permeation and bioavailability of phytoconstituents [94]. In one study, the authors prepared microspheres of zedoaryoil obtained from turmeric. The small size of the delivery system facilitated better invivo absorption and improved the bioavailability by 135.6% [94]. Further, the sustained release prevented adverse effects and reduced the dosing frequency. In another study, a nanocrystal of baicalein was formulated and the results revealed enhanced solubility and bioavailability by 1.67 times [95]. Nanotechnology has also proven beneficial to enhance the stability of essential oils. It is shown to protect essential oils from oxidation, hydrolysis, photodegradation, thermal degradation and reduce volatility. Low aqueous solubility and high volatility prevents the use of bare essential oils, making encapsulation into delivery systems a necessity [96]. Curcumin has been found to be photoreactive, which decreases its potency by 70%. Onoue et al. [73] prepared solid dispersions of curcumin to enhance the physical stability and only 17% degradation was observed. This can enhance the clinical acceptability of natural products.

Among the most important consequences of nanotechnology-based pharmaceuticals is cancer treatment, which otherwise entails many adverse effects and high costs. Certain unique innovative drug delivery methods have recently been developed using nanoparticles loaded with triclosan, which might be a turning point in preventing periodontal disease progression [97]. Peppermint oil hasbeen found to possess good anticancer properties against oral cancer; however, poor solubility limits its application. Tubtimsri et al. [98] developed a peppermint oil-loaded nanoemulsion whereby the droplet size was reduced to approx. 100 nm and further incorporation into a hydrophobic core with the exterior aqueous phase rendered them water-soluble. Further, the authors proposed the herbal shotgun approach, where synergistic combinations of peppermint oil and virgin coconut oil loaded in a nanoemulsion revealed promising cytotoxic properties against oral squamous cell carcinoma cell lines.

## 5. Role of Nano-Herbal Technology in Biofilm Resistance

Dental caries and periodontitis represent the most common oral infectious diseases. On studying the pathophysiology, it was found that invasion by pathogenic bacteria is the main etiology of the disease. These bacteria hide within the extracellular matrix, which prevents the entry of antimicrobial agents and forms biofilms. Herbal ingredients have shown activity in biofilm resistance. The main mechanism of action is preventing the synthesis of glucans, which are responsible for adherence, thus preventing the formation of biofilms [99]. Further, essential oils can play a pivotal role in the treatment of dental infections. Essential oils directly damage the integrity of cell membranes, which results in microbial growth inhibition. Infact, studies have shown that essential oils can be used as a substitute for synthetic antibacterials. For instance, the zone of inhibition against *S. aureus* and *E. coli* was found to be 9.94 ± 0.29 mm and 8.10 ± 0.31 mm, respectively, with doxycycline gel. A eugenol nanoemulsion gel was prepared and tested and the zone of inhibition was 8.82 ± 0.28 mm and 7.58 ± 0.31 mm, respectively, which is close to the antibacterial affect exhibited by doxycycline [100]. The relation between the lipophilicity of essential oils and their antimicrobial activity has driven researchers to examine the antibacterial properties of some biological components, such as *Citrus Aurantifolia*, *Thymus vulgaris*, and *Origanum vulgare* essential oils, against cariogenic oral bacteria [101]. Essential oils, due to their lipidic nature, interact with the hydrophobic bacterial cell membrane, causing the destabilization and leakage of ions, which is responsible for cell death.

However, a problem arises when the bacterial cells are protected by a hydrophilic extracellular matrix that is impermeable to essential oils. To combat the problem of resistance, nanotechnology has been successfully used, where enhanced antibacterial activity has been found [102] (Table 3). Poly(D,L-lactide-co-glycolide)(PLG) nanoparticles loaded with *H. madagascariensis* extract were prepared and tested for their antibacterial properties against Gram-positive and Gram-negative strains. The minimum bactericidal concentration (MBC) was considerably reduced for the nanoparticle formulation (1.875 × 10^2^ mg/L), compared to 5–7 × 10^2^ mg/L exhibited by extracts in ethyl acetate. The bioadhesive property of the PLG polymer allowed the attachment of nanoparticles to the bacterial cells while facilitating the controlled release of the extract and maintaining the concentration [103].

Biofilms are hydrophilic in nature, and so hydrophobic essential oils can be converted into nanoemulsions with a size of less than 300 nm, which facilitates the penetration of the active ingredient into the biofilm matrix. Cinnamon oil loaded in nanoemulsions inhibited a *S. mutans* biofilm by 86%, compared to 60% observed by an ethanolic oil solution [104]. Synergistic effects can be observed when essential oils are encapsulated in lipid-based nanodelivery systems. The nanosize facilitates higher diffusion into bacterial cell membranes. Researchers have suggested that micro-/nanoemulsions give more favorable outcomes in terms of bacterial resistance [105]. The presence of surfactants in the formulation, coupled with the nanosize, provides high surface tension and wetting ability to the delivery system. This allows fusion with the cell membranes of microorganisms and eventually kills them.

## 6. Synergistic Combinations of Phytoconstituents and Drugs in Nanotechnology

It is believed that synergistic combinations of antibiotics with herbal ingredients can potentiate the antibacterial effects, which can help to overcome bacterial resistance. A study conducted by Saquib et al. [114] suggested that the use of phytoconstituents with antibiotics is effective against periodontal infections. For instance, use of a combination of *C. zeylanicum* with azithromycin exhibited strong antibacterial activity against *T. denticola* and *T. forsythia*. A synergistic combination of *S. presica* and tetracycline showed significantly reduced MICs against most periodontal pathogens. In another study by Dera et al. [115], the efficacy of thymoquinone with different macrolide and aminoglycoside antibiotics was tested and an enhanced antibacterial effect was witnessed. It is believed that efflux pumps acting within the bacterial cells are responsible for drug resistance. Studies have shown that herbal active constituents inhibit efflux pumps and also display antibacterial activity of their own, mostly through reducing the production of acids or preventing adhesion [114,116]. Therefore, combination with antibiotics significantly enhances the antibacterial efficacy. Further details are available in Table 4.

Hydroxyapatite has been found to possess bone formation properties and is therefore used as a bone substitute in dental implants. A study has shown that hydroxyapatite nanocrystals morphologically resemble apatite crystals and promote bone remineralization, but only in the outer enamel layer [117]. In a study by Huang et al. [118], superior bone remineralization and deposition was found on teeth at a depth of 40–140 µm by using a combination of nanohydroxyapatite and Gallachinensis extracts compared to single treatments. G. chinensis is a potential anti-caries agent that favors mineralization while simultaneously inhibiting demineralization.

## 7. Regulatory and Commercial Manufacturing Challenges

The challenges for herbal ingredients associated with dental products are as follows:

- Availability of consistent quality raw material—Raw materials grown in different geographical conditions show different quality characteristics;
- Contamination with toxic or unwanted medicinal plants and/or plant parts is always present as, generally, the irrigation, collection, and supply chain are not well controlled;
- QC methods employed for herbal products are different from other conventional products, so specific expertise is required.

Furthermore, if the herbal products have been approved through the route of indigenous medicine, i.e., Ayurveda, Siddha, and Unani (ASU), they can make therapeutic claims; otherwise, they cannot be associated with any such claims. Other than ASU, dental care products fall within the categories of cosmetics (CDSCO, India), OTC drug products (USFDA), cosmetics (EMA), drug and health products (Health Canada), and cosmetics (TGA, Australia). If the product has to be marketed in the category of dietary supplements, no prior approval from the USFDA for manufacturing/selling is required. It becomes the responsibility of the manufacturer to ensure the safety of their products. The various quality attributes have to be checked by the manufacturer. The standard quality parameters of toothpaste can be obtained from the following documents:
IS 6356 (2001)Toothpaste specificationAS 2827:1982Toothpaste specificationSABS 1302:1980Toothpaste specification1S0 11609:1995 (E)Dentistry—Toothpaste requirements, test methods, and markingBS 5136:1981 SToothpaste specificationSLS 275:1980Toothpaste specificationBDS 1216:1989Toothpaste specification

The general quality parameters could be as follows:

- Fineness;
- pH of aqueous suspension;
- Heavy metal quantification (lead and arsenic);
- Foaming power;
- Fluoride quantification;
- Microbial counts (total viable counts and Gram-negative pathogens).

Similarly, other quality guidance documents can be found. However, the challenges do not end here: the standardization of herbals is another major challenge. The efficacy of a natural preparation depends on the growth conditions, collection, and processing techniques of the raw materials. Heavy metal and microbial contamination is a persistent problem if proper harvesting is not carried out. Intentional adulteration is another issue.

Stringent regulatory agencies carry out marker-based identification of raw materials. However, this remains a challenge for underdeveloped nations due to high costs. Moreover, a lack of availability of reference standards for most of the herbal ingredients makes it impractical. All these factors contribute to the poor quality of natural preparations, often leading to limited acceptability and recognition by health practitioners.

## 8. Patent Analysis

There are a number of herbal products that are available on the market for the treatment of oral diseases. However, the use of nano-herbal technology in dental diseases is a relatively new concept. A patent analysis was carried out to determine the number of patents in the area and much literature cannot be found (Table 5). Therefore, nano-herbal dentistry needs further exploration.

## 9. Future Prospects of Herbal and Essential Oil-Based Formulations in the Treatment of Dental Diseases

Phytochemical screening has already established the pharmacological properties of several biological actives. During screening studies, it was found that ingredients such as flavonoids, terpenes, and terpenoids are responsible for therapeutic effects. Invitro studies have proven that herbal remedies have potential in the treatment of dental diseases. However, the problem lies in reproducing the results invivo, which often becomes difficult. This due to the previously discussed issues, such as poor lipid solubilization and improper molecular size of herbal active molecules. In order to achieve the desired therapeutic effects of herbal ingredients, researchers are continuously working to achieve the delivery of herbal active molecules at the desired concentrations in the blood. The greatest challenge in the development of herbal formulations is to cross the membrane with an enhanced pharmacokinetic profile and therapeutic efficacy. Lipid-based and oil-based carriers can be used to resolve these challenges. Bioactive molecules with a greater half-life have a long duration of action and long rate of elimination too as compared to molecules with a shorter half-life. The elimination rate and renal filtration determine the bioavailability of herbal drugs. The greater the elimination rate and renal filtration, the lower the bioavailability of herbal drugs in blood plasma. Novel delivery systems such as nanoemulsions are used successfully to deliver herbal drug molecules to the blood at maximum therapeutic value with minimum adverse effects [122]. Moreover, encapsulation in nanodelivery systems has overcome the existing physicochemical limitations of essential oils. Sustained and controlled release systems of oils into the cells of bacteria can be achieved by attaining the chemical stability, solubility in water, and encapsulation of oils, which can enhance the antimicrobial action. Additionally, the concept of the herbal shotgun has shown a tremendous surge with the application of nanotechnology in herbal industry. Previously, simultaneously using two or more ingredients in a single formulation was difficult as the actives were incompatible with other components in the formulation. The new drug delivery systems have made it possible to improve the efficacy of natural ingredients. Additionally, constituents that were disregarded previously due to their undesired properties have now come into the fore.

Nonetheless, there are many setbacks in the nano-herbal industry. The main challenge is to scaleup the development of nanotechnology-based herbal bioactive molecules at a commercial level. Further, geographical conditions, cultivation factors, and processing conditions affect the quality and quantity of active constituents. Additionally, isolation and purification is another challenge as it is a time-consuming and costly process. Pharmaceutical industries have to collect and screen the herbal actives themselves, which is seen as an impediment and discourages the use of natural ingredients. The lack of standardization of herbal ingredients and dire agricultural practices are significant setbacks in the herbal industry. Global harmonization in regulatory guidelines related to herbal products is the need of the hour.

The global herbal medicinal market size was valued at USD 85 billion in 2019 and is expected to increase at a rate of 20%. Two new herb-based NDAs were approved by the USFDA in 2006 and 2012 for the drugs sinecatechins and crofelemer, respectively. The current challenge is to bring newer nanotechnology-based herbal products into the market with the possibility of scaling up and complying with the international standards of safety and toxicology.

## 10. Conclusions

The goal of this review was to look back over the last ten years at the possibilities of natural medicine for treating dental disorders. Abundant evidence has been found thatproves that phytoconstituents present in herbal extracts or essential oils have the potential to be used as preventative or therapeutic therapies for oral disorders. Due to various drawbacks, natural medicine has not been explored sufficiently. While herbal medicinal products are leading to new formulations, further research is required to determine their therapeutic benefits, along with their safety and efficacy. Single or combination therapies in the form of a suitable delivery system can be used to reduce the global burden of oro-dental diseases.

## Figures and Tables

**Table 1:** List of different phytoconstituents obtained from herbal sources along with their potential pharmacological activity in oro-dental diseases.

S. No	Plant	Biological Name	Active Phyto-Constitutent	Part of Plant Used	Activity	Reference
1	Neem	*Azadirachtain indica*	Azadirachtin	Leaves	Antimicrobial, anti-inflammatory,antibacterial, and antiplaque activity	[20]
2	Triphala	*Emblica officinalis*	Gallic acid, tannic acid, syringic acid, andepicatechinalong with ascorbic acid	Fruits	Antibacterial, antimicrobial, antioxidant, anti-inflammatory, and radical scavenging activity	[21]
3	Garlic	*Alliumsativum*	Allicin	Rhizomes	Antimicrobial, antibacterial, antifungal, antiviral, anti-inflammatory, and antioxidant activities	[22]
4	Gum acacia	*Acacia catechu*	Catechin, epicatechin, epigallocatechin, alkaloids, and tannins	Bark	Antibacterial, anti-inflammatory, astringent, antifungal, antimicrobial, and anticancer properties	[43]
5	Roselle	*Hibiscus sabdariffa*	Hibiscus acid andprotocatechuic acid	Seeds, leaves, fruits, and roots	Antimicrobial, antibacterial effect	[44]
6	Ginger	*Zingiber officinale*	Gingerols	Rhizome	Antimicrobial effect	[44]
7	Green tea	*Camellia sinensis*	Catechins	Dried leaves	Antibacterial activity	[44]
8	Liquorice	*Glycyrrhiza glabra*	Glycyrrhizin	Root extracts	Antiadherence, antimicrobial, and anti-inflammatory properties	[24]
9	Meswak	*Salvadora persica*	Volatile oils, flavonoids, alkaloids, steroids, terpenoids, saponins, and carbohydrates	Roots	Antibacterial, anti-inflammatory, anticariogenic	[24]
10	Turmeric	*Curcuma longa*	Curcumin	Rhizome	Analgesic, anti-inflammatory, antioxidant, antiseptic, and antimicrobial activity	[45]
11	Cinnamon oil	*Cinnamomum zeylanicum*	Cinnamaldehyde, cinnamic acid, and *trans*-cinnamaldehyde	Leaves, bark, root, and fruit	Antimicrobial activity	[46]
12	Citronella oil	*Cymbopogon nardus*	Citronellal, citronellol, nerol, geraniol, limonene	Leaves and fruit peel	Antibiofilm, antibacterial, antiseptic, antifungal, and anticariogenic activity	[47]
13	Tea tree oil	*Melaleuca alternifolia*	Terpinen-4-ol, γ-terpinene, α-terpinene	Leaves	Antimicrobial properties	[24]
14	Eucalyptus oil	*Eucalyptus globulus*	Eucalyptol, α-pinene, δ-limonene	Leaves	Antibacterial, antimicrobial, anti-inflammatory effect, andfreshening properties	[48]
15	Lemongrass oil	*Cymbopogon citratus*	Citral	Leaves	Antibacterial, antifungal, antioxidant, antiseptic, astringent, anti-inflammatory properties	[49]
16	Myrtle oil	*Myrtus* *communis*	α-pinene, limonene, 1.8-cineole, 4-terpineol, α-terpineol, linalool	Leaves	Anti-inflammatory, antimicrobial, antibacterial activity	[50]
17	Ajwain oil	*Trachyspermumammi*	Thymol, camphene, myrcene, and α-3-carene	Leavesand the seed-like fruit	Antimicrobial, antibacterial, germicidal, antifungal activity	[51]
18	Red sage	*Salvia miltiorrhiza*	Tanshinone IIA	Stem, leaves, fruit	Anticancer activity against oral squamous cancer cell line	[52]
19	Thunder duke vine	*Tripterygium wilfordii*	Triptolide	Peeled roots	Anti-inflammatory in oral lichen planus, mouth ulcers	[53]
20	Bitter bean	*Sophora alopecuroides*	Sophora alkaloids	Seeds and aerial parts	Antibacterial, anti-inflammatory	[54,55]
21	Happy tree	*Camptotheca acuminata*	Camptothecin	Bark, wood	Anticancer activity against oral squamous cancer cell line	[56]
22	Korean red ginseng	*Panax ginseng*	Ginsenosides	Root	Bone regeneration in dental implant	[57]

**Table 2:** Nanoparticle formulations of phytoconstituents with regard to dental diseases that show improved physicochemical and therapeutic properties.

Formulation	Phytoconstituent	Source	Outcome	Reference
Nanosuspension	Zerumbone	*Zinigiber zerumbet* rhizome	Formulations with 200 nm particle size were prepared, which significantly (*p* < 0.05) enhanced the saturation solubility and dissolution 2-fold	[71]
Inclusion complex with hydroxylpropyl-β-cyclodextrin	Zerumbone	*Zinigiber zerumbet* rhizome	Enhanced the solubility >30-fold	[72]
Nanoemulsion	Curcumin	*Curcuma longa* rhizomes	The droplet size of the formulation was 196 nm, which enhanced the dissolution by upto 95% and bioavailability 8-folds	[73]
	Tanshinone IIA	Root of *Salvia miltiorrhiza*	Smaller particle size (95.6 nm) enabled faster dissolution, 100% in 20 min, and better cytotoxic properties can be expected	[74]
Nanoparticles	Tanshinone IIA	*Radix salvia miltiorrhiza*	Small size of the nanoparticles improved the dissolution of tanshinone and better bioavailability can be expected	[63]
	Berberine	*Berberis aristata*	Encapsulation into nanoparticles reduced the crystallinity of berberine coupled with small size, which significantly (*p* < 0.0001) enhanced the aqueous solubility and dissolution. The antimicrobial activity also increased 3–4-fold against Gram-positive bacteria, Gram-negative bacteria, and yeasts	[75]
Phytosomes	Epigallocatechin-3-gallate	*Thea sinensis*	Complexation with phospholipids helped in increasing oral absorption and plasma drug concentration 2-fold, which suggests its potential in enhancing bioavailability	[76]
	Silybin	Silymarin	The phospholipid complex augmented the lipophilicity of silymarin and improved the oral bioavailability 4-fold	[76]
Ethosomes	Lemannine, matrine, sophoridine, sophocarpine	*Sophora alopecuerides*	Loading sophora alkaloids in ethosomes provided penetration to deeper skin layers (up to 180 µ) and facilitated transdermal delivery, which is a viable alternative to avoid bitter taste of drug	[77]
	Curcumin	*Curcuma longa*	Ethosomes were prepared with 93% entrapment efficiency. The formulation enhanced skin permeation, which suggests that it can be used for transdermal delivery. High rate of metabolism in intestine and rapid clearance can be overcome by transdermal delivery of curcumin	[78]
Microspheres	Camptothecin	*Camptotheca acuminata*	Camptothecin is sensitive to pH changes in the body. Encapsulation in PLGA microspheres provided stability through acidic microenvironment. The size of the microspehers (1.3 µm) improved antitumor activity by enhancing uptake by cancer cells	[79]
	*Ginsenosides*	Ginseng	Chitosan microspheres provided adhesion to bone cells and the active compound ginsenosides promoted bone regeneration	[80]
Microemulsion	Elemene oil	*Curcuma wenyujin*	Microemulsion improved the aqueous solubility, stability, and oral bioavailability (163%) of the volatile oil	[81]
	Triptolide	*Tripterygium wilfordii*	The formulation provided sustained and prolonged delivery of herbal ingredient which is useful for limiting the toxicity associated with drug	[82]
Solid lipid nanoparticles	Curcumin	*Curcuma longa*	SLN improved the solubility and bioavailability of curcumin and thus MIC and MBC wereconsiderably reduced	[83]
	Triptolide	*Tripterygium wilfordii*	SLN loaded with triptolide was taken up by lymphatic system and exhibited negligle toxicity to liver and kidney. Improved anti-inflammatory activity due to increase in oral bioavailability and prolonged plasma drug levels was observed	[84]
Liposomes	Silymarin	*Silybum marianum*	Silymarin hybrid liposomes were developed to improve its poor bioavailability. It showed improved hepatoprotective activity, enhanced permeation through buccal mucosa, and stability of silymarin	[85]
	Garlic oil	*Allium sativum*	SLN were prepared with >90% entrapment efficiency. The formulation also improved the solubility of garlic oil, as evident by drug relase studies carried out in phosphate-buffered medium (11% in 17 h)	[86]
	Curcumin	*Curcuma longa*	Encapsulation in liposomes increased the solubility and anti-inflammatory activity in 2-hydroxyethyl methacrylate induced inflammation in dental pulp stem cells	[87]
Self- nanoemulsified delivery system (SNEDDS)	Matrine	*Sophora flavescens*	Matrine was complexed with phospholipid and lipid solubility was increased by 600%. Further, the complex was loaded in SNEDDS, increasing the intestinal absorption and ultimately oral bioavailability by 60%	[88]

**Table 3:** Nanodelivery systems of phytoconstituents and their role in microbial biofilm resistance.

Phytoconstituent	Nanodelivery System	Bacterial Sp.	Outcome	Reference
Nano punica granatum and nano garlic herbal extract	Nanoemulsification	*Enterococcus faecalis* and *Staphylococcus epidermidis*	Significantly (*p* < 0.001) higher dead bacterial count was witnessed withnano-herbal extracts when compared to medicated calcium hydroxide gel. Insignificant differences were observed between pomegranate and garlic extract.	[106]
Eugenol	Nanoemulsion	*S. aureus* and *E. coli*	The eugenol nanoemulsion gel showed improved antibacterial activity (double) compared to eugenol solution. The small size helped in fusion with bacterial cells and the surfactants in the formulation disrupted the cell membrane.	[100]
Cinnamon, clove	Silver nanoparticles	*Streptococcus mutans*	Cinnamon and clove silver nanoparticles exhibited wider zones of inhibition (10 mm) compared to amoxycillin (8 mm), suggestive of good antibacterial efficiency.	[107]
*Syzygium cumini*	Silver nanoparticles	*C. albicans* and *S. mutans*	The extracts encapsulated in silver nanoparticles exhibited improved antimicrobial properties, as suggested by a ratio of MIC of 0.98 for silver nanoparticles to seed extracts.	[108]
*Mentha* spp.	Solid lipid nanostructure	*Streptococcus mutans* and *Streptococcus pyogenes*	The findings demonstrated that *Mentha* essential oil loaded in nanostructure increased theantibacterial activity (zone of inhibition 20 mm, compared to 10 mm shown by essential oil solution).	[109]
Tea tree oil	Nanoparticles	*P. aeruginosa*	Tea tree oil nanoparticles reduced the motility of bacteria (by 62%) and adhesion of biofilms, which was otherwise not detected on using bare oil.	[110]
Tea tree oil	Nanoparticles	*P. gingivalis*, *A. actinomycetemcomitan*, *F. nucleatum*	Nanoparticles were prepared with size of 198 nm. Small size allowed penetration within the biofilm matrix and the bacterial viability was 26%, compared to 51% shown by *M. alternifolia* oil.	[111]
Lemongrass oil (Citral)	Chitosan nanoparticles	Gram-positive and Gram-negative bacteria	Chitosan nanoparticles increased the thermal stability of oil. The antimicrobial properties increased sinificantly (*p* < 0.001) when compared to bare oil.	[112]
Lemongrass oil	Nanocapsule	*P. aeruginosa*, *E. coli*, *C. albicans*, *S. aureus*	The lemongrass oil reduced the MIC by almost half when loaded in nanocapsules. The biofilm formation was also reduced by 2 times for all the species except *P. aeruginosa*.	[113]
Eucalyptus oil (eucalyptol, α-pinene, and δ-limonene)	Nanoemulsion	*P. aeruginosa*, *Candida* spp.		

**Table 4:** Studies showing potential of synergistic combinations of herbal ingredients and synthetic drugs in dental diseases.

Formulation	Drug	Phytoconstitutent	Outcome	Reference
Nanoparticles	Chlorhexidine	*Scutellaria baicalensi*	Study showed one-fold enhanced antibacterial effects of nanoparticles with chlorhexidine and *Scutellaria baicalensi* (MIC 50 µg/mL) on oral bacterial biofilms compared to either treatment used alone (MIC 100 µg/mL).	[119]
Liposome	Lauric acid	Curcumin	Liposome formulation containing lauric acid and curcumin in 1:1 ratio exhibited 1.5–2-fold greater antibacterial activity than their single forms.	[120]
Nanostructured lipid carriers	Ampicillin	Curcumin	The formulation showed synergistic antibacterial efficacy and enhanced the wound healing rate.	[121]

**Table 5:** A snapshot of patents highlighting the use of nanotechnology in herbal dentistry.

Patent No.	Published	Description
U.S. 10,342,840 B2	9 July 2019	Titanium dioxide nanomaterials adsorbed with organic functional groups and citric acid herbal extracts for antimicrobial activity
WO 2021/116917 A1	17 June 2021	Nanocellulose with active herbal ingredients formulated as gels/films

## Data Availability

Not applicable.
